# Supplementary figures and images for: Redescription of four Epiperipatus species with an update on the distribution of Epiperipatus acacioi (Marcus & Marcus, 1955)
Source: PeerJ. 2025 Apr 28;13:e19168. doi: 10.7717/peerj.19168 (PMC12045270; doi:10.7717/peerj.19168)

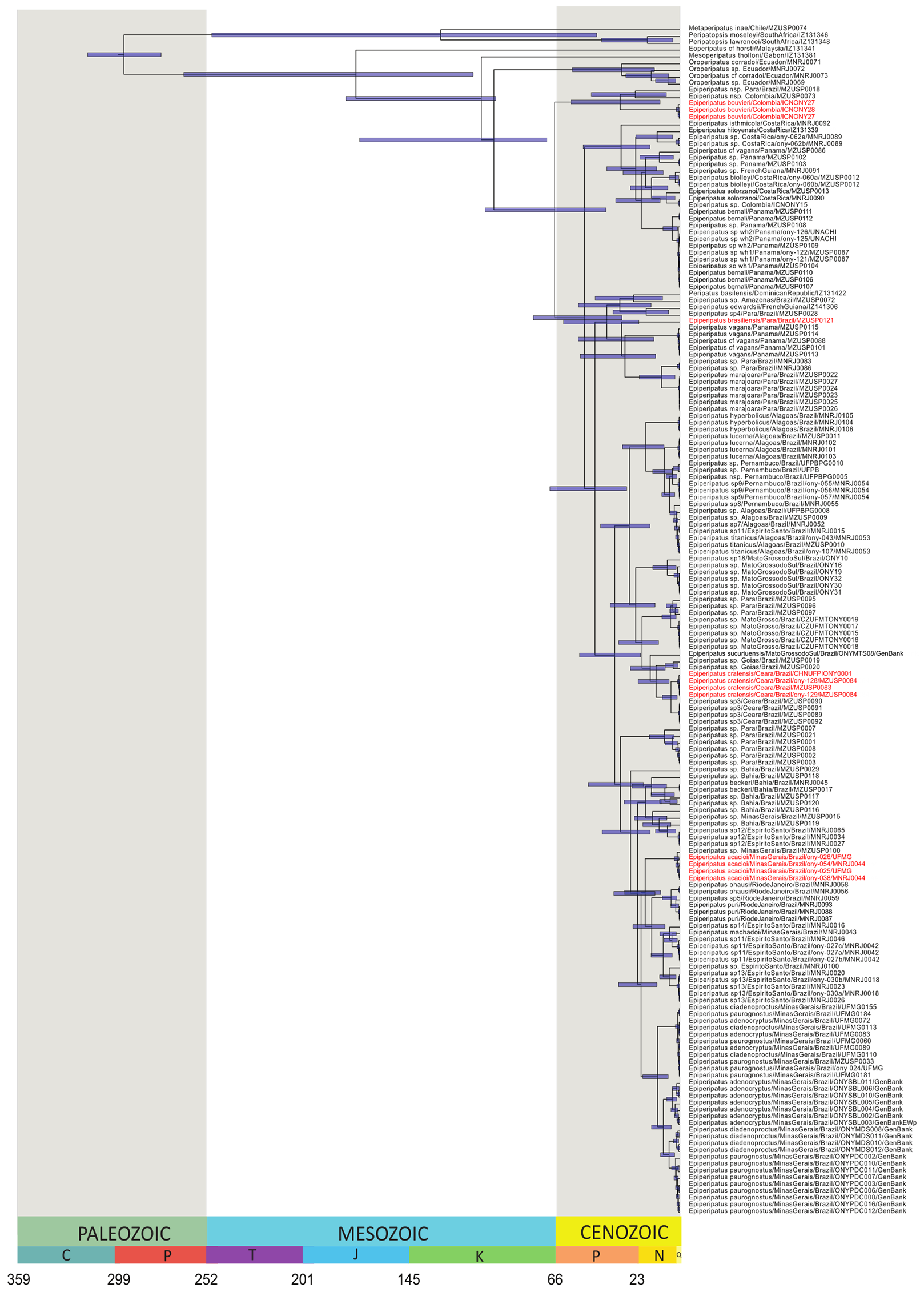

Supplement: Supplemental Information 1 — In Neopatida, the terminals in red indicate Epiperipatus acacioi, E. bouvieri, E. brasiliensis, and E. cratensis. [file peerj-13-19168-s001.png]

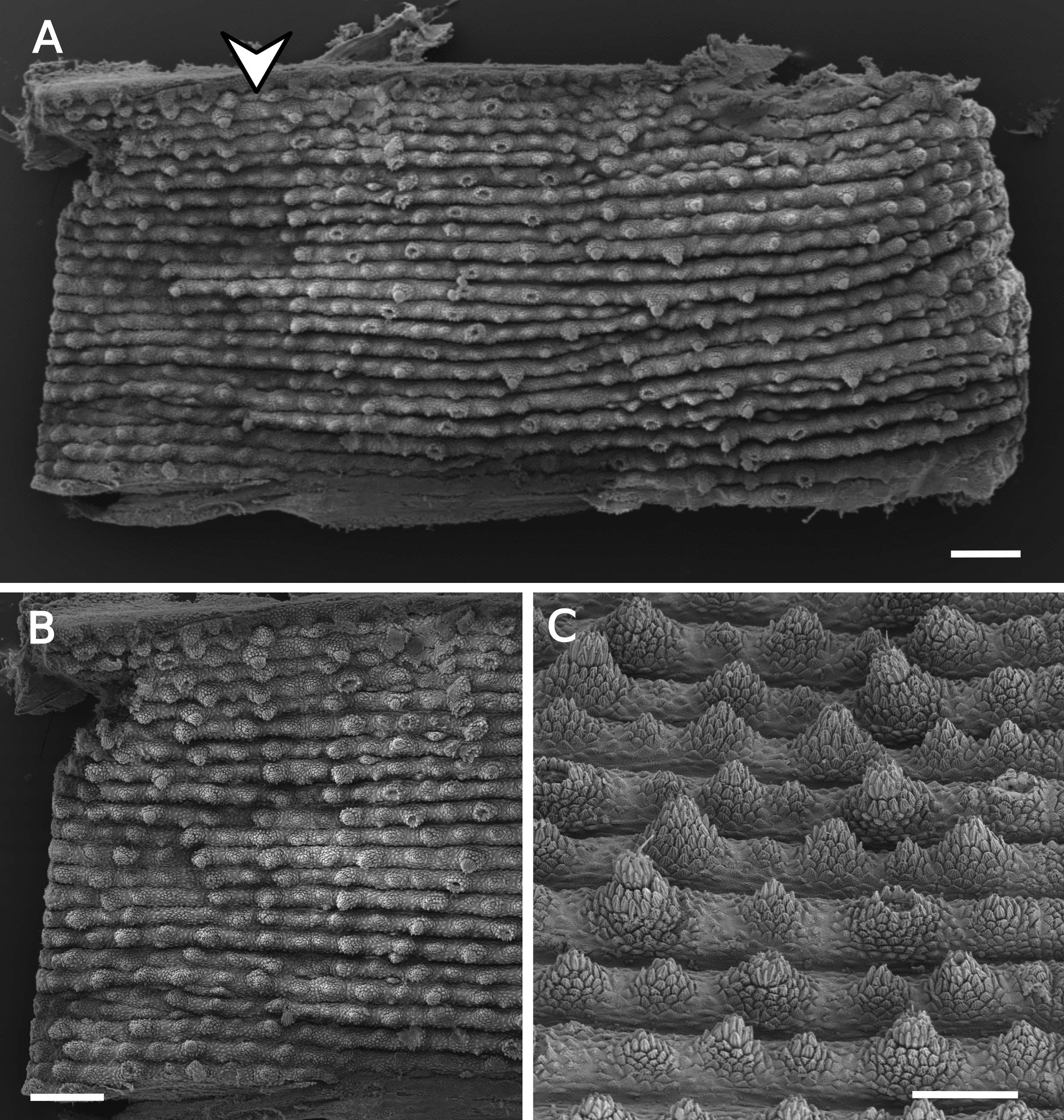

Supplement: Supplemental Information 2 — (A) Section of the dorsal integument corresponding to one segment in the middle of the body; note the arrowhead indicating the dorsomedian furrow. (B) Detail of the dorsomedian showing the absence of light organs. (C). Dorsal papillae arrangement of the holotype, highlighting the primary papilla varying in size and absence of dorsal papillae on dorsal furrows. Scale bars A and B = 200 µm, C = 100 µm. [file peerj-13-19168-s002.png]

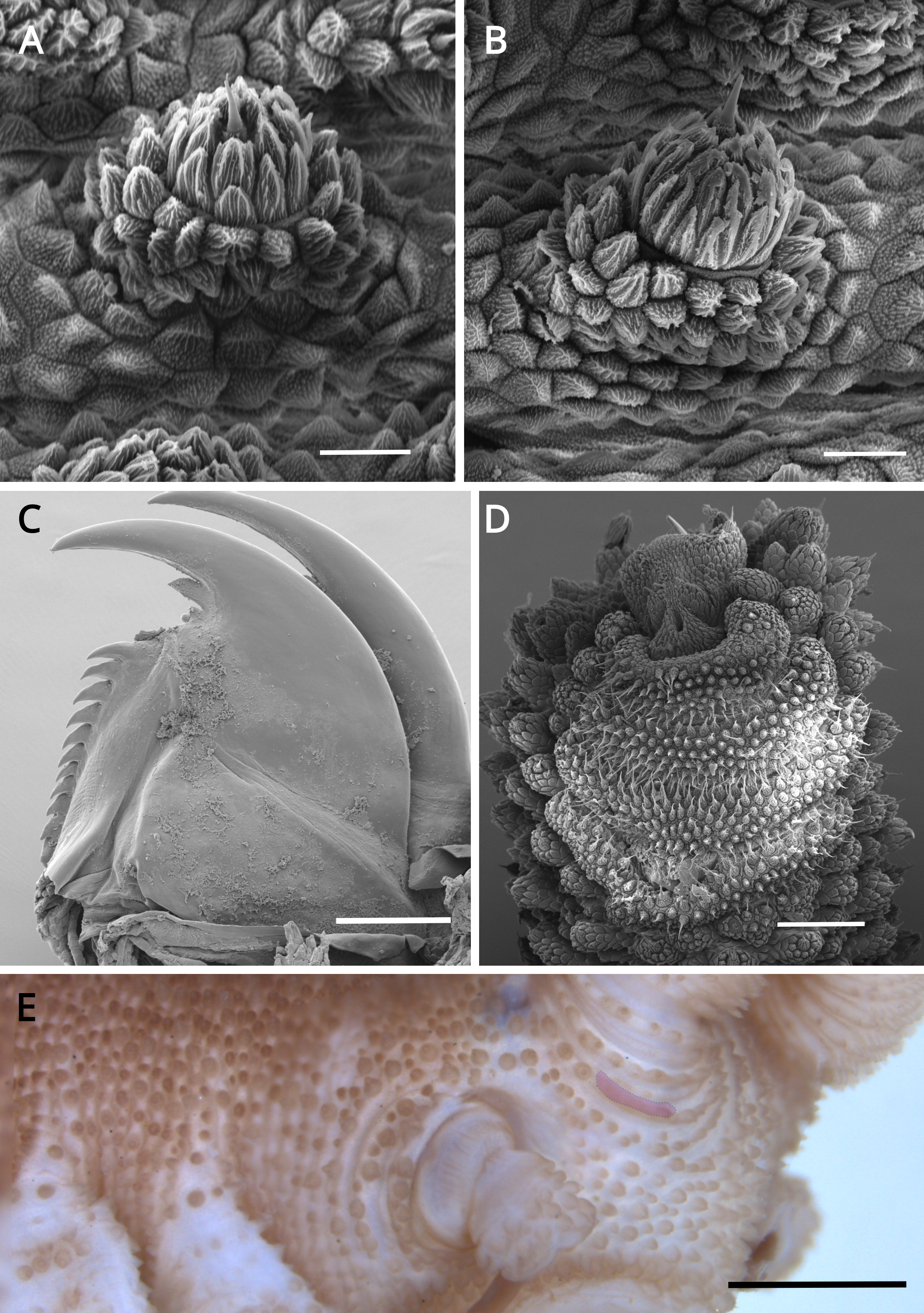

Supplement: Supplemental Information 3 — (A) Primary papillae in prolateral view, and (B) in retrolateral view. (C) Jaw with inner blade foreground covering the accessory tooth of outer jaws. (D) Spinous pad and nephridial tubercles. (E) Frontal organ (highlighted with a dashed line and purple background) on the right side. Scale bars A and B = 20 µm, C and D = 100 µm, E = 500 µm. [file peerj-13-19168-s003.png]
